# Optimal Molecular Methods in Detecting p190^**BCR-ABL**^ Fusion Variants in Hematologic Malignancies: A Case Report and Review of the Literature

**DOI:** 10.1155/2015/458052

**Published:** 2015-04-08

**Authors:** Rebecca J. Sonu, Brian A. Jonas, Denis M. Dwyre, Jeffrey P. Gregg, Hooman H. Rashidi

**Affiliations:** ^1^Department of Pathology and Laboratory Medicine, University of California Davis Medical Center, Sacramento, CA 95817, USA; ^2^Department of Internal Medicine, Division of Hematology and Oncology, University of California Davis Medical Center, Sacramento, CA 95817, USA

## Abstract

Patients with BCR-ABL1 positive hematologic malignancies and Philadelphia-like B-lymphoblastic leukemia (B-ALL) are potential candidates for targeted therapy with tyrosine kinase inhibitors (TKI). Before TKIs, patients with B-ALL had a much worse prognosis and current treatments with targeted TKI therapy have improved outcomes. Thus, the detection of BCR-ABL1 is crucial and a false negative BCR-ABL1 result may adversely affect patient care. We report a case of a 76-year-old male with a new diagnosis of B-ALL who was initially found to be BCR-ABL1 negative by quantitative polymerase chain reaction (PCR). A concurrent qualitative PCR was performed which detected a positive BCR-ABL1 result that was confirmed by a next generation sequencing (NGS) based assay and identified as the rare fusion variant e1a3 of p190^BCR-ABL^. Based on this result, the patient was placed on dasatinib as a targeted therapy. In the era of molecular diagnostic medicine and targeted therapy, it is essential to have an understanding of the limitations of molecular assays and to follow a comprehensive diagnostic approach in order to detect common abnormalities and rare variants. Incorporating NGS methods in an algorithmic manner into the standard diagnostic PCR-based approach for BCR-ABL1 will aid in minimizing false negative results.

## 1. Introduction

The Philadelphia chromosome (Ph), t(9;22)(q34;q11.2), is consistently seen in chronic myelogenous leukemia (CML), 25% of adult B-acute lymphoblastic leukemia (B-ALL), 2–4% of childhood B-ALL, and rarely in acute myeloid leukemias [1]. The Ph chromosome results from the breakpoint in the BCR gene on chromosome 22, which usually occurs in the major breakpoint cluster region (M-bcr) between exons e12–e16 (also known as b1–b5). The breakpoints in the ABL gene on chromosome 9 occur usually at exon a2. From these breakpoints, the resulting fusion transcripts are known as e13a2 (b2a2) and e14a2 (b3a2) [2]. These fusion transcripts are then translated into a 190-kDa protein, p190^BCR-ABL^, encoded by the fusion transcript e1a2 in the minor breakpoint region (m-bcr) which is commonly associated with B-ALL in both adults and children. The 210-kDa protein known as p210^BCR-ABL^ is most commonly seen in CML [2]. Ultimately, these alternative chimeric BCR-ABL1 proteins produced are responsible for the oncogenic properties of these hematologic neoplasms. Additionally, due to alternative splicing, other transcript variants from the major and minor breakpoint regions can occur with or independently of the common fusion transcripts described above.

We report a case of a 76-year-old male with a new diagnosis of B-ALL that was initially found to be BCR-ABL1 negative by quantitative PCR. A concurrent qualitative PCR was also performed which was able to detect a low level positive BCR-ABL1 result which was subsequently confirmed by a next generation sequencing (NGS) based assay and identified as the rare BCR-ABL1 fusion variant e1a3 of p190^BCR-ABL^. Based on this positive result, the patient was ultimately placed on dasatinib as a targeted therapy.

## 2. Case Report

### 2.1. Clinical History and Pathology

**Figure 1:**
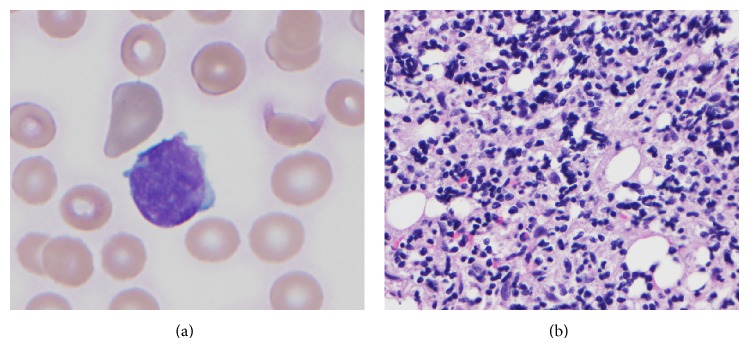
B-Lymphoblastic leukemia. (a) Peripheral blood (Wright-Giemsa, 100x) showing a circulating large sized blast. (b) Bone marrow biopsy (hematoxylin & eosin, 50x) showing fibrotic marrow with small area of immature mononuclear cells. No areas of normal hematopoiesis were seen.

A 76-year-old man with a history of coronary artery disease, chronic obstructive pulmonary disease, prostate cancer in remission, tobacco and alcohol abuse, and Schizoaffective disorder was admitted after being found to have a platelet count of 10 K/mm^3^ (normal 130–400 K/mm^3^). He was significantly fatigued and had a petechial rash. After transfusion of a unit of platelets, his complete blood count showed a white blood cell count (WBC) of 7.1 K/mm^3^ (normal 4.5–11 K/mm^3^), hemoglobin 13.1 g/dL (normal 13.5–17.5 g/dL), platelets of 34 K/mm^3^, and a leukoerythroblastic peripheral blood smear showing 2% circulating blasts (Figure 1(a)). Computed tomography showed mild splenomegaly but no distinct lymphadenopathy was noted. Other studies, including a metabolic panel, liver function tests, coagulation panel, and viral serologies, were negative. The patient had no known previous history of CML or peripheral blood findings suggestive of CML.

A subsequent bone marrow biopsy showed a hypercellular marrow (approximately 85% of total cellularity) with 95% of the cellularity involved by diffuse sheets of monotonous round to slightly irregular lymphoblasts (Figure 1(b)). The biopsy showed marked reticulin fibrosis. Immunohistochemical stains performed on the core biopsy showed blasts that were positive for CD34, CD79a, CD10, PAX-5, TdT, and CD20. Flow cytometry showed that the blasts were positive for dim CD45, CD34, CD10, CD19, CD20 (dim), HLA-DR, CD79a, TdT, CD22 (subset), and IgM and negative for myeloperoxidase (MPO), CD13, CD33, and CD117. Overall, these findings were consistent with a B-lymphoblastic leukemia.

**Figure 2:**
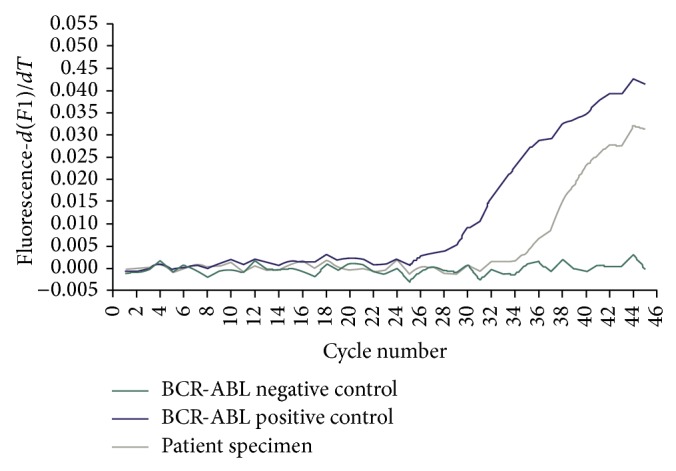
BCR-ABL1 qualitative RT-PCR amplification curves. Lightcycler amplification curves showing the cycle number of the crossing point (CP), the point where the reaction's fluorescence reaches the maximum of the second derivative of the amplification curve corresponding to the point where the acceleration of the fluorescence signal is at its maximum. The CP is 29 cycles for the positive control (purple), 35 cycles for the patient's peripheral blood specimen (grey), and no amplification for the negative control (no DNA) (green). Thus, the BCR-ABL1 fusion transcript was detected from the patient's peripheral blood.

Molecular studies for the BCR-ABL1 mutation showed a low positive qualitative PCR result for the BCR-ABL1 fusion transcript (Figure 2). A repeat qualitative PCR test reconfirmed the low positive result. However, the quantitative PCR testing for the BCR-ABL1 fusion transcripts (p190 and p210 isoforms) yielded negative results. Due to this discrepancy, peripheral blood was sent for testing on the FoundationOne Heme assay (Foundation Medicine, Cambridge MA), a next generation sequencing (NGS) based assay. Targeted RNA-seq found 17 transcripts for the variant e1a3 p190^BCR-ABL^ fusion. DNA was sequenced to a median coverage depth of 492x. No other fusion transcripts were identified including p210^BCR-ABL^ or p230^BCR-ABL^. Previous validation experiments confirmed the sensitivity for detection of fusion events as >99% for tumor cell fractions between 20 and 50% and 97% for tumor cell fractions of 10%. High specificity was confirmed for all fusion events (PPV > 95%) [3]. In addition, a PTPN11 V428M mutation of unclear significance was also detected. Cytogenetics and FISH studies failed to yield results mainly due to the lack of cell growth, as per the outside performing institution. T- and B-cell gene rearrangement studies were not performed.

Due to the positive BCR-ABL1 result, the patient was started on induction therapy with dasatinib and steroids as per the protocol described by Foà et al. [4]. One month after starting therapy, the patient's counts improved (WBC count was 8.7 K/mm^3^ with normal differential, hemoglobin was 10.9 g/dL, and platelets were 171 K/mm^3^) and he became transfusion independent. He tolerated the treatment well and by Day 98 of dasatinib treatment, the patient continued to be in complete hematologic remission. Monitoring for the BCR-ABL1 transcript will be performed with the qualitative PCR testing and if negative with the NGS assay.

## 3. Materials and Methods

The qualitative BCR-ABL1 RNA test was performed at the University of California Davis Medical Center Molecular Pathology Laboratory (Sacramento, CA). The Lightcycler assay detects the BCR-ABL1 fusion transcripts b3a2, b2a2, and e1a2, which covers more than 95% of the described t(9;22) translocations [5]. The HybProbe chemistry in the Roche LightCycler BCR-ABL1 t(9;22) kit was used. This probe format uses two labeled oligonucleotides, one with a fluorescent dye at the 3′ terminus and the other with a different dye at the 5′ end. The probes are designed to hybridize to the BCR-ABL1 target strand, such that both dyes are in close proximity. Therefore, one dye acts as donor fluorophore, whereas the other (acceptor) emits light if it is positioned near the donor dye. By using this probe chemistry in the LightCycler, the acceptor fluorescence emission is measured during the annealing step when both probes hybridize to the BCR-ABL1 target strand.

**Figure 3:**
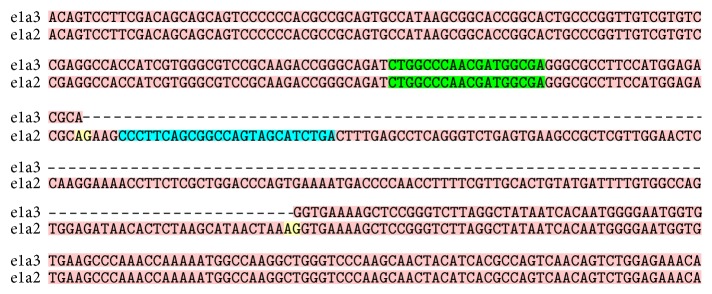
Sequence alignments between e1a2 (p190) and the variant e1a3 (p190) on a portion of the BCR-ABL1 sequence. Example of specific primers [6] (green and blue) used to detect e1a2 with the quantitative RT-PCR method. Notice that the exon a2 sequence (blue) is lacking in the e1a3 transcript, represented by the dotted line. The lack of this primer binding site sequence results in a negative BCR-ABL1 p190 result with the p190 quantitative PCR test.

The quantitative BCR-ABL1 fusion tests were performed at ARUP Laboratories (Salt Lake City, UT) utilizing reverse-transcriptase-quantitative PCR (RT-qPCR) for the detection of BCR-ABL1 transcripts e1a2 (or p190) and e13a2 and e14a2 (or p210). Total RNA is isolated and converted to cDNA. Fusion transcripts are quantitated by real time PCR amplification. The primers are designed to detect the major (p210) BCR-ABL1 breakpoint including fusions between BCR exon 13 and ABL1 exon 2 (e13a2) and BCR exon 14 and ABL1 exon 2 (e14a2) and the minor (p190) BCR-ABL1 breakpoint with a fusion between BCR exon 1 and ABL1 exon 2 (e1a2) (Figure 3). The targeted portions of the BCR-ABL1 variant sequences of interest were retrieved from the NCBI (National Center for Biotechology Information) database and were aligned using the sequence alignment tool, T-COFFEE version 11.00. An example of the primer sequences that are depicted in Figure 3 was obtained from literature [6].

FoundationOne Heme Assay (Foundation Medicine, Cambridge MA) uses hybridization capture applied to ≥50 nanograms of extracted DNA or RNA for 405 cancer related genes and select intronic regions from 31 genes (FoundationOne Heme DNA only, *n* = 405); targeted RNA-seq for rearrangement analysis was performed for 265 genes that are frequently rearranged in cancer. Sequencing of captured libraries was performed using an Illumina HiSeq 2500 to a median exon coverage depth of >250x, and resultant sequences were analyzed for base substitutions, insertions, deletions, copy number alterations (focal amplifications and homozygous deletions), and select gene fusions, as previously described. To maximize mutation-detection accuracy (sensitivity and specificity) in impure clinical specimens, the test was previously optimized and validated to detect base substitutions at a ≥5% mutant allele frequency (MAF) and indels with a ≥10% MAF with ≥99% accuracy [3].

## 4. Discussion

We report a 76-year-old male with a recent diagnosis of B-ALL with the rare fusion variant e1a3 of p190^BCR-ABL^, confirmed by next generation sequencing. The e1a3 BCR-ABL1^p190^ variant fusion transcript lacks the ABL1 exon a2 and results in a protein that lacks the N-terminal two-thirds of the Src homology 3 (SHE3) domain. The domain is required for full leukemogenic potential* in vivo* and its absence has been proposed to be associated with a more benign clinical course [7, 8]. According to a literature review, the e1a3 variant has only been reported in a few cases of B-ALL and CML (Table 1) [7–19]. Fujisawa et al. have recognized a poor prognosis in all B-ALL cases as well as those in CML blast phase; however, the clinical significance or phenotype of this variant has yet to be determined [13]. Verma et al. have suggested that CML patients with BCR-ABL1^p190^ need to be identified as high-risk patients and monitored closely for efficacy during TKI therapy [2]. Overall the clinical significance in the detection of this or any other variant of BCR-ABL1 is that a positive result will ultimately lead to a different course of management, follow-up, and treatment.

Rare BCR-ABL1 fusion transcript variants can potentially go undetected with the use of limited molecular assays such as the quantitative RT-PCR method that targets specific fusion sequences with limited primers. For example, primers are selected specifically for e1a2 (for p190) and e13a2 and e14a2 (for p210) (Figure 3). Therefore, the detection of variants such as e1a3 could be missed with the standard quantitative PCR method given the absence of exon a2 (Figure 3) [15]. In our case, a qualitative RT-PCR method, that reportedly detects 95% of the described t(9;22) translocations [5], identified a low positive result in a patient with a reported negative quantitative PCR test (Figure 2). This low positive qualitative PCR result may have been due to the low blast percentage (2% of white blood cells) in the peripheral blood specimen. But overall, it has led to the suspicion of a possible BCR-ABL1 mutation and subsequent testing with a next generation sequencing based assay which confirmed the BCR-ABL1 transcript and ultimately identified the e1a3 of p190^BCR-ABL^ variant.

Ultimately, a qualitative BCR-ABL1 test can aid in the detection of a rare BCR-ABL1 fusion variant when confirmed with a next generation sequencing based assay that is designed to provide a high throughput assessment on clinically relevant mutations that influence therapeutic decisions. Furthermore, this case illustrates that NGS based assays are more sensitive than the traditional PCR approach and may be very useful in the workup of suspected BCR-ABL1 neoplasms in certain circumstances. In addition, NGS would also be able detect Ph-like B-ALL cases, which are BCR-ABL1 negative and characterized by a gene-expression profile similar to that of BCR-ABL1 positive B-ALL with genetic alterations that are also responsive for TKIs [20].

We propose the following approach for molecular testing for BCR-ABL1 for the detection of BCR-ABL1 at the time of diagnosis to optimize the detection of rare fusion variants which could enhance patient care. First, perform qualitative PCR with cytogenetics and/or FISH testing. If positive, perform quantitative PCR p190/p210 to determine the BCR-ABL1 transcript. If negative for quantitative PCR/cytogenetics testing or equivocal for qualitative PCR testing, proceed to next generation sequencing to detect possible rare variant transcripts and/or Ph-like ALL disease. This will allow the capture of all clinically significant molecular mutations that could potentially guide therapy and management.

One limitation in detecting rare variant transcripts solely via NGS is the inability to use standard PCR, in some cases, for residual disease monitoring and follow-up. Therefore, NGS may be the only alternative for determining the presence or absence of residual disease.

## 5. Conclusion

In the era of molecular diagnostic medicine and a repertoire of potential targeted therapy options, it is essential to have a thorough understanding of the limitations of the individual molecular assays and to follow a sound yet comprehensive diagnostic approach that would not only detect the common abnormalities but rather be able to find the rare variants as well. Incorporating next generation sequencing methods into our current standard diagnostic PCR approach for BCR-ABL1 and BCR-ABL1 negative Ph-like B-ALL will aid in minimizing false negative results and may ultimately enhance patient care. In addition, with the higher specificity of testing at the sequencing level, the rare e1a3 variant of BCR-ABL1^p190^ may not actually be as infrequent as previously thought. The actual clinical significance of these rare variants is not yet fully understood and their role in clinical management with targeted therapy needs to be further elucidated with the aid of detecting variants and molecular profiles by newer molecular methods such as next generation sequencing.

## Figures and Tables

**Table 1:** Published cases of e1a3 fusion variants in BCR-ABL1 associated hematologic neoplasms [7–19].

Age/sex	BCR-ABL1 transcript	Disease classification	Patient's clinical status	Citation
39/f	e1a3	B-ALL	Dead	Soekarman et al. [9]
1/f	e1a3	B-ALL	Dead	Iwata et al. [10]
62/f	e1a3	B-ALL	Dead	Langabeer et al. [11]
—	e1a3	B-ALL	—	Wilson et al. [12]
25/f	e1a3	B-ALL	Dead	Fujisawa et al. [13]
48/m	e1a3	B-ALL	Dead	Burmeister et al. [8]
61/f	e1a3	B-ALL	Alive	Burmeister et al. [8]
45/f	e1a3	B-ALL	Alive	Burmeister et al. [8]
64/f	e1a3	B-ALL	Dead	Burmeister et al. [8]
31/m	e1a3	B-ALL	Alive	Burmeister et al. [8]
78/f	e1a3	B-ALL	Alive	Chen et al. [14]
34/m	e1a3	B-ALL	Alive	Shin et al. [15]
17/m	e1a3	B-ALL	Dead	Shin et al. [15]
76/m	e1a3	B-ALL	Alive	Current case
41/m	e1a3 + e1a2	CML	Alive	Al-Ali et al. [7]
64/f	e1a3	CML	Alive	Al-Ali et al. [7]
42/m	e1a3	CML	Alive	Goh et al. [16]
75/f	e1a3	CML	Alive	Roman et al. [17]
68/f	e1a3 + b2a3	CML	Alive	Martinelli et al. [18]
80/m	e1a3	CML, B-ALL^∗^	Alive	Martinez-Serra et al. [19]

B-ALL, B-lymphoblastic leukemia/lymphoma; CML, chronic myeloid leukemia; f, female; m, male. ^∗^CML with B-ALL type blast crisis.
